# Clinical Evaluation of INNO-LiPA HPV Genotyping *EXTRA* II Assay Using the VALGENT Framework

**DOI:** 10.3390/ijms19092704

**Published:** 2018-09-11

**Authors:** Lan Xu, Elizaveta Padalko, Anja Oštrbenk, Mario Poljak, Marc Arbyn

**Affiliations:** 1 Unit of Cancer Epidemiology, Belgian Cancer Centre, Sciensano (Previously Scientific Institute of Public Health), 1050 Brussels, Belgium; lan.xu@sciensano.be; 2 Department of Clinical Chemistry, Microbiology and Immunology, Ghent University Hospital, 9000 Gent, Belgium; elizaveta.padalko@uzgent.be; 3 Institute of Microbiology and Immunology, Faculty of Medicine, University of Ljubljana, 1000 Ljubljana, Slovenia; Anja.Ostrbenk@mf.uni-lj.si (A.O.); Mario.Poljak@mf.uni-lj.si (M.P.)

**Keywords:** INNO-LiPA, VALGENT, HPV genotyping, cervical cancer, human papillomavirus, clinical validation, Hybrid Capture 2, Linear Array

## Abstract

In this diagnostic test validation study, we assessed the clinical accuracy and HPV genotyping performance of the INNO-LiPA HPV Genotyping *Extra* II (INNO-LiPA) within the VALGENT-3 framework. VALGENT is designed to assess the analytical and clinical performance of HPV tests with genotyping capacity. The VALGENT-3 panel comprised 1300 consecutive cervical cell specimens enriched with 300 samples with abnormal cytology obtained from women attending the Slovenian cervical cancer screening programme. The INNO-LiPA allows type-specific detection of 32 HPV types; however, for the clinical accuracy assessment, we considered it as high-risk (hr)HPV positive when at least one of the following HPV types was present: HPV16, HPV18, HPV31, HPV33, HPV35, HPV39, HPV45, HPV51, HPV52, HPV56, HPV58, HPV59, and HPV68. Clinical accuracy for detection of cervical intraepithelial neoplasia grade 2 or worse (CIN2+) was compared between INNO-LiPA and Hybrid Capture 2 (HC2), which is a standard comparator test for HPV tests used in cervical cancer screening. In addition, hrHPV and type-specific detection HPV types were compared between INNO-LiPA and Linear Array HPV Genotyping Test (Linear Array). The prevalence of hrHPV determined by INNO-LiPA was 17.1% (95% CI, 15.0–19.2%) in the screening population. HrHPV testing with INNO-LiPA had a sensitivity for CIN2+ of 96.9% (95% CI, 92.1–99.1%) which was non-inferior to HC2 (relative sensitivity of 1.01; 95% CI, 0.97–1.04; *p*_n.inf_ = 0.0002) and a specificity for ≤CIN1 of 85.3% (95% CI, 83.2–87.3%) which was inferior to HC2 (relative specificity of 0.95; 95% CI, 0.93–0.97; *p*_n.inf_ = 0.9998). Genotyping agreement between INNO-LiPA and Linear Array was excellent for hrHPV, HPV16, HPV18, HPV35, HPV45, HPV58 and HPV59, but good or fair for other HPV types. To conclude, INNO-LiPA demonstrated non-inferior clinical sensitivity but lower specificity compared to HC2 in addition to excellent concordance compared to Linear Array for hrHPV and some genotypes.

## 1. Introduction

Over 200 human papillomavirus (HPV) types have been identified and classified based on their nucleotide sequences, with new HPV types being characterized at an increasing rate [1]. Among them, 12 high-risk HPV (hrHPV) types (HPV16, HPV18, HPV31, HPV33, HPV35, HPV39, HPV45, HPV51, HPV52, HPV56, HPV58 and HPV59) are causally linked with cervical cancer and their immediate precursors [2]. In addition, eight more HPV types have been associated with some rare cases of cervical cancer (HPV26, HPV53, HPV66, HPV67, HPV68, HPV70, HPV73 and HPV82) [3]. The recognition of the strong etiological association between persistent hrHPV infection and cervical cancer has led to the development of novel HPV tests to enhance secondary prevention of the cervical cancer [4,5]. Furthermore, randomized controlled trials (RCTs) have demonstrated that HPV-based screening is more effective than cervical cytology in reducing the incidence of invasive cervical carcinoma in primary screening for cervical cancer for women aged 30 years or older [5,6]. Thus, a number of countries are currently in the process of switching from cervical cytology to HPV based primary screening for cervical cancer [7].

Many HPV tests are available on the market but only few have been clinically validated for use in primary screening settings [8]. The Hybrid Capture 2 assay (HC2; Qiagen, Hilden, Germany) [6,9,10,11] and GP5+/6+ PCR-based enzyme immunoassay (GP5+/6+-EIA; Diassay, Rijwijk, the Netherlands) [12,13,14] are HPV DNA assays that had been clinically validated for primary screening based on longitudinal evidence obtained from large RCTs. Therefore, HC2 and GP5+/6+-EIA are accepted as the standard comparator tests in evaluations of alternative HPV tests [15]. Several other HPV tests have been fully or partially validated and demonstrate non-inferior clinical sensitivity and specificity for detection of cervical intraepithelial neoplasia grade 2 or worse (CIN2+) compared to the standard comparator tests and high inter-and intra-laboratory reproducibility [15,16]. Majority of validated HPV tests target 13 or 14 hrHPV types in aggregate, but some have limited (partial genotyping for HPV16 and HPV18 only), extended (separate genotyping of HPV16, HPV18 and other hrHPV types) and full (type-specific genotyping of all included types) genotyping ability [16]. Since HPV16 and HPV18 are responsible for approximately 70% of cervical cancer, partial HPV genotyping for these two types is frequently used in the triage of HPV-positive women [17,18]. Although the usefulness of full genotyping of hrHPV types is not yet established, a recent study showed that, in addition to HPV16, HPV31 and HPV33 are more carcinogenic than other hrHPV types, suggesting that wider genotyping may also be clinically valuable [19].

INNO-LiPA HPV genotyping assay, based on the principle of reverse hybridization after highly sensitive PCR amplification with SPF10 primers, have been used for HPV genotyping over two decades [20,21]. During this timeframe, the original assay has undergone several modifications, resulting in a few different versions. The INNO-LiPA HPV Genotyping *Extra* II assay (INNO-LiPA; Fujirebio Europe, Ghent, Belgium) evaluated in the current study is the most recent assay launched by the company in 2015, targeting 32 types, four types more than the previous version. This new version contains genotype specific probes for more decisive genotyping results, an upgraded SPF10 primer set resulting in improved sensitivity (comparable for all hrHPV types), improved human DNA control primers and provides a ready-to-use amplification reagent [22].

In the present study, the VALidation of HPV GENotyping Tests (VALGENT) framework was used to evaluate the clinical accuracy of INNO-LiPA in comparison with HC2. For the first time, it was verified whether hrHPV testing with INNO-LiPA fulfills the minimal requirements for use in primary cervical cancer screening [15]. In addition, type-specific concordance was compared between the INNO-LiPA and the Linear Array HPV Genotyping Test (Linear Array; Roche Molecular Diagnostics, Branchburg, NJ, USA). HrHPV testing with the latter test was recently clinically validated through the VALGENT network as well and has been proposed as a standard analytical HPV genotyping comparator test to resolve discordant typing results of clinically validated HPV assays [23].

## 2. Results

The characteristics of the VALGENT-3 study population, including demographics, cytological and histological results have been described previously [23,24]. Of the 1600 samples analyzed by INNO-LiPA, four samples showed no signal for human HLA-DPB1 gene control line. These four samples were considered as invalid and therefore excluded for further analysis. Of the 1296 valid samples obtained from screening population, 17.1% women (221/1296) tested positive for the presence of any of the 13 hrHPV types by INNO-LiPA. The overall and type-specific prevalence of 13 hrHPV types in the total study population determined by INNO-LiPA is summarized in Table 1. The hrHPV prevalence was 15.2% in women with NILM (negative for intraepithelial lesion or malignancy) and increased to 42.8%, 69.0% and 86.0% in women diagnosed with ASC-US (atypical squamous cells of undetermined significance), LSIL (low-grade squamous intraepithelial lesion) and HSIL(high-grade squamous intraepithelial), respectively. The risk ratio (RR) of HSIL compared to women with NILM was highest (RR > 8.00) in women infected with in HPV16, HPV33, HPV18 and HPV45.

### 2.1. Clinical Performance of the INNO-LiPA

The accuracy data for the INNO-LiPA and HC2 for the outcomes CIN2+, CIN3+ and ≤CIN1 are shown in Table 2 for the total study population and for women aged 30 years or older. When the whole study population was considered, INNO-LiPA detected 123 of 127 CIN2+ cases and 81 of 82 CIN3+ cases, which corresponds to a sensitivity of 96.9% (95% CI, 92.1–99.1) and 98.8% (93.4–100), respectively. The specificity for ≤CIN1 of INNO-LiPA (1034/1212) was 85.3% (95% CI, 83.2–87.3). Similar results were obtained for women aged 30 years or older.

The relative sensitivity of INNO-LiPA compared to HC2 was 1.01 (95% CI, 0.97–1.04; *p*_mcn_ = 0.6547; *p*_n.inf_ = 0.0002) for CIN2+ and 1.01 (95% CI, 0.97–1.06; *p*_mcn_ = 0.5637; *p*_n.inf_ = 0.001) for CIN3+. The relative specificity of INNO-LiPA for ≤CIN1 was 0.95 (95% CI, 0.93–0.97; *p*_mcn_ = 0.0000; *p*_n.inf_ = 0.0000). Similar results were obtained if the analysis was restricted to women ≥30 years (Table 3).

### 2.2. Genotyping Agreement between INNO-LiPA and Linear Array

In the total study population, concordance between INNO-LiPA and Linear Array was assessed at type-specific level and overall for 13 hrHPV types (Table 4). Overall concordance of the two assays for 13 hrHPV types was 93.0% and the corresponding κ value was 0.805 (95% CI, 0.757–0.0854), indicating excellent agreement between INNO-LiPA and Linear Array. Similarly, the level of agreement was also excellent for detection of HPV16, HPV18, HPV35, HPV45, HPV58 and HPV59. However, for the identification of other individual types, level of agreement ranged from good to poor between the two assays (Table 4). In addition, INNO-LiPA detected more positive cases than Linear Array for all individual types common to both assays.

## 3. Discussion

The INNO-LiPA provides full genotyping capability for 32 HPV types. For the purpose of this study, INNO-LiPA was considered positive if at least one of the 13 hrHPV types targeted by HC2 was detected.

To the best of our knowledge, the INNO-LiPA has not been validated previously according to the international guidelines for evaluation of new HPV tests in primary cervical cancer screening settings. Here, we present the first study to evaluate the clinical performance of the INNO-LiPA compared to HC2 using samples from the VALGENT-3 panel. In the whole study population, the INNO-LiPA showed a sensitivity for the detection of CIN2+ and CIN3+ of 97% and 99%, respectively, which was similar to HC2. However, the clinical specificity for ≤CIN1 was only 85%, which was 5% (95% CI, 3–7%) lower than the comparator test.

INNO-LiPA is a SPF10 PCR that targets a short highly conserved region in the L1 gene [21,26]. The small size of the amplicon makes the test analytically very sensitive. However, at the same time, discrimination of the individual types is challenging and complex and it is therefore not so surprising that the clinical specificity is lower compared to HPV tests targeting longer DNA sequences [27]. The small size of the amplicon makes INNO-LiPA particularly useful for testing of archived cell preparations or formaline-fixed-paraffin-embedded tissue blocks stored over long periods where parts of the viral genome can be fragmented [28].

INNO-LiPA provides for each HPV type a qualitative output, which is translated into a positive/negative result. The appreciation of presence or absence of blue lines is not quantifiable. Therefore, adaptation of the cut-off, which may allow a more optimal balance between clinical sensitivity and specificity, is in case of INNO-LiPA not possible.

Excellent analytical agreement between INNO-LiPA and Linear Array was observed for 13 hrHPV types overall, HPV16, HPV18, HPV35, HPV45, HPV58 and HPV59. In addition, INNO-LiPA detected more positive cases than Linear Array for all individual HPV types that are common to both assays, with a positivity rate of hrHPV determined by INNO-LiPA statistically significantly higher than that determined by Linear Array (*p*_mcn_ < 0.001) in the total study population. The significant difference of positivity rate for hrHPV with these two genotyping methods is consistent with the tests’ clinical performances with regard to relative specificity compared to HC2.

In conclusion, in our study, INNO-LiPA exhibits lower clinical specificity; however, this is most likely due to detection of HPV infections with low concentrations and therefore can play an important role in evaluation of viral infection outcomes of vaccination trials and in monitoring the impact of HPV vaccination. Moreover, INNO-LiPA may be useful in epidemiological studies to investigate the prevalence and distribution of HPV types and in studies of the natural history of HPV infection at the type-specific level.

## 4. Materials and Methods

### 4.1. Clinical Specimens

The VALGENT framework is designed to assess the comparative analytical and clinical performance of HPV tests that offer limited to full genotyping capability [29]. VALGENT is iterative, using specimens collected in different countries. The first two VALGENT panels have been completed, using samples collected from Belgium [30,31,32] and Scotland [33,34,35,36]. The third study panel (VALGENT-3) was collated in Slovenia as previously described [11,23,24]. Briefly, 1300 consecutive cervical samples were collected from women who participated in the organised Slovenian national cervical cancer screening program between December 2009 and August 2010 (screening population). The study panel was enriched with 300 cytologically abnormal specimens collected between January 2014 and May 2015 (enrichment population). As required in the VALGENT protocol [29], the enrichment population included 100 women with atypical squamous cervical cells of undetermined significance (ASC-US), 100 women with low-grade squamous intraepithelial lesion (LSIL) and 100 women with high-grade squamous intraepithelial lesion (HSIL).

The sample collection, aliquoting procedure and storage details have been described in detail in previous VALGENT-3 manuscripts [23,24]. Briefly, in July 2016, Ghent University (Ghent Belgium) received 1600 samples of extracted DNA. DNA was extracted from original samples collected into ThinPrep PreservCyt solution (Hologic, Marlborough, MA, USA). Prior to DNA extraction, 1 mL aliquot of original ThinPrep sample was centrifuged at 13,000× *g* for 15 m with supernatant being discarded and cellular pellet resuspended in 200 µL PBS buffer. DNA extraction was performed using QIAamp MinElute Media Kit (Qiagen, Hilden, Germany) following the manufacturer’s instructions. Bound DNA was finally eluted with 50 µL of ATE buffer and stored at −70 °C prior to further testing. According to the manufacturer’s instructions, we have used 10 µL of extracted DNA for INNO-LiPA testing. Similarly, from the second aliquot of original ThinPrep DNA was extracted for Linear Array testing, where 50 µL of extracted DNA was used for further testing.

### 4.2. HPV Testing

#### 4.2.1. INNO-LiPA HPV Genotyping *Extra* II

The INNO-LiPA is a line probe assay based on the principle of reverse hybridization for qualitative detection and identification of 32 different HPV types, including 13 hrHPV (HPV16, HPV18, HPV31, HPV33, HPV35, HPV39, HPV45, HPV51, HPV52, HPV56, HPV58, HPV59 and HPV68), 6 possible hrHPV (HPV26, HPV53, HPV66, HPV70, HPV73 and HPV82), 9 low-risk HPV (HPV6, HPV11, HPV40, HPV42, HPV43, HPV44, HPV54, HPV61 and HPV81) plus 4 other HPV genotypes (HPV62, HPV67, HPV83 and HPV89). INNO-LiPA uses the biotinylated consensus primers (SPF10) to amplify a 65-bp region within the L1 region of multiple alpha HPV types. The resulting biotinylated amplicons are then denatured and hybridized with specific oligonucleotide probes. A primer set for the amplification of the human HLA-DPB1 gene is included to monitor sample quality and extraction. The INNO-LiPA assay (sample incubation, stringent wash and color development) was performed fully automated using the AutoBlot 3000H (Bio-Rad Laboratories Inc., Hercules, CA, USA). Interpretation of the developed strips was done by scanning and automated interpretation using with the LiRAS for LiPA HPV software (Version 3.01, Fujirebio Europe, Ghent, Belgium). The test was performed in accordance with the manufacturer’s instructions.

#### 4.2.2. HC2

HC2 detects 13 hrHPV types (HPV16, HPV18, HPV31, HPV33, HPV35, HPV39, HPV45, HPV51, HPV52, HPV56, HPV58, HPV59, and HPV68) and is accepted as a standard comparator test for the clinical validation of hrHPV DNA assays that may be used for primary cervical cancer screening [15]. For the purpose of the present study, hrHPV positivity for INNO-LiPA was defined as the presence of one or more of the 13 hrHPV targeted by HC2.

#### 4.2.3. Linear Array

The Linear Array is an HPV test with full genotyping capacity, which detects 37 high- and low-risk HPV types (HPV6, HPV11, HPV16, HPV18, HPV26, HPV31, HPV33, HPV35, HPV39, HPV40, HPV42, HPV44, HPV45, HPV51, HPV52, HPV53, HPV54, HPV56, HPV58, HPV59, HPV61, HPV62, HPV64, HPV66, HPV67, HPV68, HPV69, HPV70, HPV71, HPV72, HPV73, HPV81, HPV82, HPV83, HPV84, HPV89, and IS39) that is frequently used in virological and epidemiological research. In the present study, Linear Array is used as a comparator test to evaluate the analytical genotyping accuracy of the INNO-LiPA.

### 4.3. Clinical Outcome and INNO-LiPA Performance Assessment

As described in previous VALGENT-3 reports [23,24], cytological assessment and referral of patients with abnormal cytology results to colposcopy were done according to the Slovenian national screening guidelines [37], which are in agreement with European guidelines [38]. Colposcopy-directed punch biopsies were obtained from suspicious areas for final histopathological assessment.

Women with histologically confirmed CIN2+ results were considered as diseased subjects. Due to the fact that women with normal cytological results of negative for intraepithelial lesion or malignancy (NILM) were not referred to colposcopy verification in our study, we considered them as subjects without disease only if they had two or more consecutive NILM cytological results (at enrolment and at subsequent screening between 12 to 48 months later). We used this group of women to compute the clinical specificity for ≤CIN1.

The clinical sensitivity and specificity of the INNO-LiPA for CIN2+ and CIN3+ were calculated. We compared the clinical accuracy of INNO-LiPA to HC2 for CIN2+ and CIN3+, using non-inferiority statistics with a relative sensitivity threshold of 90% and a relative specificity threshold of 98% [39]. The McNemar statistic was used in order to compare the differences between matched proportions [40]. For both statistics, the level of significance was set at 0.05. All analyses were performed using STATA version 14 (Manufacturer, College Station, TX, USA).

Separate and consensus genotyping agreement for the types common to INNO-LiPA and Linear Array was assessed using κ [41] and McNemar statistics [40]. κ values from 0.0 to 0.20, 0.21 to 0.40, 0.41 to 0.60, 0.61 to 0.80 and 0.81 to 1.0 indicate poor, fair, moderate, good and excellent level of agreement between two assays. A McNemar *p*-value of <0.05 indicates significant discordance between genotyping results determined by the two assays.

## Figures and Tables

**Table 1:** Overall prevalence of hrHPV (aggregate of 13 types) and of individual hrHPV types detected by INNO-LiPA in the total study population according to baseline cytology.

HPV Type	HrHPV Prevalence (No. and %) by Cytology Results				Ratio Prevalence HSIL/NILM
	NILM (*N* = 1234)		ASC-US (*N* = 131)	LSIL (*N* = 113)	HSIL (*N* = 114)
13 hrHPV *	187 (15.2%)	56 (42.8%)	78 (69.0%)	98 (86.0%)	5.7
HPV16	32 (2.6%)	12 (9.2%)	27 (23.9%)	56 (49.1%)	18.9
HPV18	12 (1.0%)	4 (3.1%)	9 (8.0%)	10 (8.8%)	8.8
HPV31	54 (4.4%)	22 (16.8%)	19(16.8)	23 (20.2%)	4.6
HPV33	11 (0.9%)	5 (3.8%)	9 (8.0%)	11 (9.7%)	10.8
HPV35	3 (0.2%)	1 (0.8%)	0 (0.0%)	2 (0.9%)	4.5
HPV39	16 (1.3%)	1 (0.8%)	5 (4.4%)	2 (1.8%)	1.4
HPV45	6 (0.5%)	5 (3.8%)	4 (3.5%)	5 (4.4%)	8.8
HPV51	31 (2.5%)	4 (3.1%)	9 (8.0%)	5 (4.4%)	1.8
HPV52	27 (2.2%)	10 (7.6%)	11 (9.7%)	7 (6.1%)	2.8
HPV56	11 (0.9%)	2 (1.5%)	7 (6.2%)	5 (4.4%)	4.9
HPV58	9 (0.7%)	3 (2.3%)	7 (6.2%)	5 (4.4%)	6.3
HPV59	11 (0.9%)	3 (2.3%)	4 (3.5%)	0 (0.0%)	0
HPV68	16 (1.3%)	5 (3.8%)	7 (6.2%)	5 (4.4%)	3.4

NILM, negative for intraepithelial lesion or malignancy; ASC-US, atypical squamous cells of undetermined significance; LSIL, low-grade squamous intraepithelial lesion; HSIL, high-grade squamous intraepithelial. * A positive hrHPV result represents detection of at least one of the 13 hrHPV types included in the HC2: HPV16, HPV18, HPV31, HPV33, HPV35, HPV39, HPV45, HPV51, HPV52, HPV56, HPV58, HPV59 and HPV68. Women infected with multiple HPV types were counted only once.

**Table 2:** Sensitivity of INNO-LiPA and HC2 for detection of CIN2+ and CIN3+ and specificity of both assays for detection of ≤CIN1. Analysis was performed separately for the total study population and for women ≥30 years old.

Assay, Study Population and Clinical Outcome	Sensitivity			Specificity		
	*n*/*N*	%	95% CI	*n*/*N*	%	95% CI
INNO-LiPA ^a^						
Total study population						
CIN2+	123/127	96.9	(92.1–99.1)			
CIN3+	81/82	98.8	(93.4–100)			
≤CIN1				1034/1212	85.3	(83.2–87.3)
Women >30 years old						
CIN2+	95/98	96.9	(91.3–99.4)			
CIN3+	65/66	98.5	(91.8–100)			
≤CIN1				887/1009	87.9	(85.7–89.9)
**HC2**						
Total study population						
CIN2+	122/127	96.1	(91.1–98.7)			
CIN3+	80/82	97.6	(91.5–99.7)			
≤CIN1				1092/1212	90.1	(88.3–91.8)
Women >30 years old						
CIN2+	94/98	95.9	(89.9–98.9)			
CIN3+	64/66	97.0	(89.5–99.6)			
≤CIN1				935/1009	92.7	(90.9–94.2)

^a^ Positive INNO-LiPA results represents detection of at least one of the following 13 hrHPV types included in the HC2: HPV16, HPV18, HPV31, HPV33, HPV35, HPV39, HPV45, HPV51, HPV52, HPV56, HPV58, HPV59 and HPV68. *n*, number of cases; *N*, total number of cases; CI, confidence interval.

**Table 3:** Relative sensitivities for detection of CIN2+ and CIN3+ and relative specificity for detection of ≤CIN1 of INNO-LiPA versus HC2. Analysis was performed separately for the total study population and for women ≥30 years old.

INNO-LiPA vs. HC2	Relative Sensitivity	Relative Specificity	*p*_mcn_^a^	*p*_n.inf_^b^
Total study population				
CIN2+	1.01 (0.97–1.04)		0.6547	0.0002
CIN3+	1.01 (0.97–1.06)		0.5637	0.001
≤CIN1		0.95 (0.93–0.97)	<0.001	0.9998
Women > 30 years old				
CIN2+	1.01 (0.96–1.06)		0.6547	0.001
CIN3+	1.02 (0.96–1.07)		0.5637	0.003
≤CIN1		0.95 (0.93–0.97)	<0.001	0.999

^a^*p* for the McNemar test for a difference between matched proportions and *p*_mcn_ > 0.05 indicates that the sensitivity or specificity of the INNO-LiPA assay are not significantly different from that of the HC2. ^b^ *p* for the test for non-inferiority. A sensitivity threshold of at least 90% and a specificity threshold of at least 98% relative to that of the HC2 were applied in a non-inferiority score test. *p*_n.inf_ < 0.05 means that the sensitivity or specificity of the INNO-LiPa is not significantly lower than that of the HC2.

**Table 4:** Agreement (concordance and κ values) between the INNO-LiPA and the Linear Array for overall hrHPV positivity and for 29 individual HPV types common to both assays in the total study population.

HPV Type	I+/L+	I+/L−	I−/L+	I−/L−	Concordance	κ (95% CI)	*p*_mcn_^a^
13 hrHPV ^b^	318	103	9	1166	93.0%	0.805 (0.757–0.854)	<0.001
HPV16	112	16	2	1466	99.0%	0.920 (0.871–0.969)	0.001
HPV18	31	6	3	1556	99.4%	0.870 (0.822–0.920)	0.3173
HPV31	68	50	1	1417	96.8%	0.712 (0.664–0.759)	<0.001
HPV33	24	13	1	1558	99.2%	0.770 (0.722–0.818)	0.0013
HPV35	5	0	0	1591	100.0%	1.000 (0.951–1.049)	1.0000
HPV39	15	9	2	1570	99.1%	0.728 (0.680–0.777)	0.0348
HPV45	14	6	0	1576	99.6%	0.822 (0.774–0.870)	0.0143
HPV51	33	16	1	1546	98.9%	0.790 (0.742–0.838)	<0.001
HPV52	30	26	2	1538	94.6%	0.674 (0.633–0.714)	0.8840
HPV56	16	9	7	1564	99.0%	0.662 (0.613–0.712)	0.6171
HPV58	19	5	0	1572	99.7%	0.882 (0.833–0.931)	0.0253
HPV59	17	2	2	1575	99.8%	0.894 (0.845–0.943)	1.0000
HPV68	7	26	0	1563	98.4%	0.345 (0.308–0.382)	<0.001
HPV26 ^c^	0	0	0	1596	100.0%	-	1.0000
HPV53	43	23	1	1529	98.5%	0.774 (0.726–0.822)	<0.001
HPV66	29	16	1	1550	98.9%	0.768 (0.720–0.816)	<0.001
HPV70	11	10	0	1575	99.4%	0.685 (0.638–0.731)	0.0016
HPV73	19	7	2	1570	99.4%	0.788 (0.739–0.837)	0.0956
HPV82	5	4	1	1586	99.7%	0.665 (0.617–0.713)	0.1797
HPV06	7	14	1	1574	99.1%	0.479 (0.435–0.523)	0.0008
HPV11	2	3	0	1591	99.8%	0.571 (0.526–0.615)	0.0833
HPV40	1	4	0	1591	99.8%	0.333 (0.296–0.369)	0.0455
HPV42	3	5	9	1579	99.1%	0.296 (0.248–0.344)	0.2850
HPV54	10	14	12	1560	98.4%	0.427 (0.378–0.476)	0.6949
HPV61	16	17	6	1557	98.6%	0.575 (0.527–0.623)	0.0218
HPV62	15	12	9	1560	98.7%	0.582 (0.533–0.632)	0.5127
HPV67	3	5	1	1587	99.6%	0.498 (0.452–0.543)	0.1025
HPV81	3	3	1	1589	99.8%	0.599 (0.551–0.647)	0.3173
HPV83	0	6	3	1587	99.4%	−0.003 (−0.049–0.044)	0.3173

I+ = INNO-LiPA positive; I− = INNO-LiPA negative; L+ = Linear Array positive; L− = Linear Array negative. Color legend (adapted from Landis and Koch for the levels of agreement [25]): dark green (1.00 ≥ κ >0.80): excellent; light green (0.80 ≥ κ > 0.60): good; yellow (0.60 ≥ κ > 0.40): moderate; orange (0.40 ≥ κ > 0.20): fair; red (0.20 ≥ κ > 0.00): poor. ^a^ *p* for the McNemar test for a difference between matched proportions and *p*_mcn_ < 0.05 indicates that the HPV positivity detected by INNO-LiPA is significantly different from that of the Linear Array. ^b^ 13 hrHPV types: HPV16, HPV18, HPV31, HPV33, HPV35, HPV39, HPV45, HPV51, HPV52, HPV56, HPV58, HPV59 and HPV68. ^c^ No HPV26 positive cases detected by both assays, κ value not applicable.

## Abbreviations

INNO-LiPA	INNO-LiPA HPV Genotyping *Extra* II assay
HC2	Hybrid Capture 2
Linear Array	Linear Array HPV Genotyping Test
HPV	Human papillomavirus
hrHPV	High-risk HPV
RCT	Randomised controlled trials
GP5+/6+-EIA	GP5+/6+ PCR-based enzyme immunoassay
VALGENT	VALidation of HPV GENotyping Tests
CIN	Cervical intraepithelial neoplasia
CIN2+	CIN grade 2 or worse
CIN3+	CIN grade 3 or worse
ASC-US	Atypical squamous cells of undetermined significance
LSIL	Low-grade squamous intraepithelial lesion
HSIL	High-grade squamous intraepithelial lesion
NILM	Negative for intraepithelial lesion or malignance
